# A Band-Pass Filter Based on Half-Mode Substrate Integrated Waveguide and Spoof Surface Plasmon Polaritons

**DOI:** 10.1038/s41598-019-50056-9

**Published:** 2019-09-17

**Authors:** Lei Zhao, Yuan Li, Zhao-Min Chen, Xin-Hua Liang, Jun Wang, Xiaopeng Shen, Qingfeng Zhang

**Affiliations:** 1 School of Information and Control Engineering, China University of Mining and Technology, Xuzhou, 221116 China; 2 Center for Computational Science and Engineering, School of Mathematics and Statistics, Jiangsu Normal University, Xuzhou, Jiangsu 221116 China; 3 School of Physics and Electronic Engineering, Jiangsu Normal University, Xuzhou, Jiangsu 221116 China; 4 State Key Lab of Millimeter-Waves, School of Information Science and Engineering, Southeast University, Nanjing, 210096 Jiangsu China; 5 Department of Physics, China University of Mining and Technology, Xuzhou, 221116 China; 6 Department of Electrical and Electronic Engineering, Southern University of Science and Technology, Shenzhen, China

**Keywords:** Electronics, photonics and device physics, Electrical and electronic engineering

## Abstract

In this paper, a band-pass filter based on half-mode substrate integrated waveguide (HMSIW) and double-layer spoof surface plasmon polaritons (SSPPs) consisting of two corrugated metal strips is proposed, which can realize band-pass transmission by etching periodic grooves at the top and bottom metal layers of the HMSIW. Moreover, the influences of important parameters on the performance of the proposed band-pass filter are analyzed by parametric study. By changing the key parameters, the low and high cut-off frequency can be controlled independently. The corresponding equivalent circuit of the proposed band-pass filter is put forward to explain the physical mechanism. Compared with the previous structures, this structure features smaller size, wider bandwidth and lower loss. Simulated results show that the proposed band-pass filter achieves a bandwidth (for |*S*_11_| < −10 dB and |*S*_21_| > −0.8 dB) of about 69.77% (15.6–32.1 GHz). The measured results have good agreements with the simulated ones, which verify that the proposed band-pass filter has good performances and potential applications at the microwave frequencies.

## Introduction

Substrate integrated waveguide (SIW) is a competitive transmission structure that has received extensive attentions^1,2^. The upper and lower layers of the basic SIW structures are metal layers, and the middle layer is a low loss dielectric layer. Metallic via holes arrays are formed on both sides of the metal coated dielectric substrate, and electromagnetic waves are confined in a rectangular cavity formed by metallic via holes arrays and two metal layers. The propagation characteristics of a SIW are similar to a rectangular waveguide^3^, which has a low cut-off frequency. Additionally, the low cut-off frequency can be controlled by changing the width of SIW. In the past decades, the concept of half-mode substrate integrated waveguide (HMSIW) was proposed that retains the characteristics of the SIW and reduces the size by half^4,5^. HMSIW has the advantages of light weight, small size, low loss, and easy integration. Therefore, it has been widely used in microwave transmission, such as filters^6,7^ and antennas^8,9^.

Surface plasmon polaritons (SPPs) are the surface electromagnetic waves propagating along the interface between medium and metal at visible or ultraviolet (UV) frequencies, exponentially decaying in the direction perpendicular to the interface. However, at lower frequencies, such as microwave and terahertz frequencies, the metal behaves as perfect electrical conductor (PEC). Therefore, SPPs cannot be excited in the microwave frequency range. The spoof surface plasmon polaritons(SSPPs) which can support a kind of SPP-like surface waves overcome the limitation that SPPs do not occur in the microwave frequencies^10–12^, thus SSPPs have attracted great attention and achieved a lot of achievements^13–15^. In additional, SSPPs have the advantages of controlling their dispersion characteristics and cut-off frequency by tuning the structural parameters of the metal^16–21^. Hence, SSPPs have widely applied in the microwave frequencies^22,23^. An ultrathin metallic structure printed on a dielectric substrate was proposed^24^ to achieve a broadband band-pass filter with a low loss in the microwave frequency band. It shows that the subwavelength arrays of grooves which can support SSPPs mode and SSPPs have an excellent transmission property. A double-layer SSPPs structure was proposed^25^, which demonstrates that the double layers SSPPs structure have a higher transmission efficiency than a single layer.

Considering the low-pass features of SSPPs structure and high-pass features of SIW structure, the first hybrid SSPP-SIW filter was proposed^7^. Recently, a band-pass filter based on the SSPPs and SIW was proposed^26^. But the filter using SIW combined with SPPs has a larger size, which is not easy for integration. Meanwhile, a hybrid SIW-SPP transmission structure was proposed^27^, where SPPs propagate through arrays of transverse metallic blind holes that were designed inside the SIW. However, it needs a thicker substrate. Recently, a hybrid HMSIW-SSPPs filter was proposed^28^, which overcomes the shortcomings of large size. However, its transmission efficiency needs to be further improved.

In this paper, we propose a band-pass filter based on HMSIW and double-layer SSPPs, which can realize band-pass transmission by etching periodic grooves on the top and bottom metal layers of HMSIW. Compared with the previous structures, our design has the following features: (1) it has higher transmission efficiency and wider bandwidth; (2) the low and high cut-off frequencies can be controlled by changing related parameters; (3) the small and simple design is easier to fabricate and has potential applications at the microwave frequencies; (4) an equivalent circuit of the proposed filter is put forward to explain the physical mechanism.

## Results

### Design configuration

**Figure 1:**
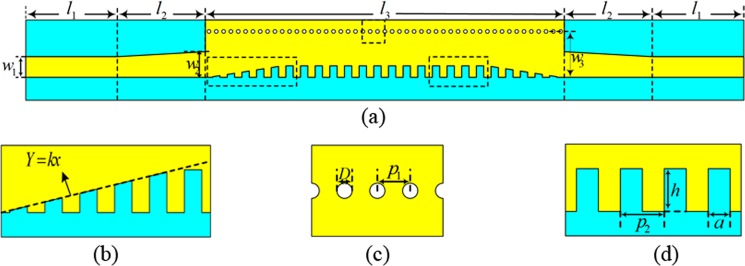
Configuration of the proposed band-pass filter. (**a**) Top view (The bottom view is the same of the top view); (**b**) the gradient grooves; (**c**) metallic via holes; (**d**) periodic grooves.

The configuration of the proposed band-pass filter is presented in Fig. 1(a), which is composed of three parts: (1) a 50 Ω microstrip line, (2) a conversion structure, and (3) a HMSIW with periodic grooves for transporting SSPPs to realize the band-pass performance. The blue parts in Fig. 1 are flexible thin dielectric F4B substrate (relative permittivity *ε*_*r*_ = 2.65, loss tangent tan*δ* = 0.0015) whose thickness is 0.5 mm. The yellow parts in Fig. 1 stand for metal (copper) and its thickness is 0.018 mm. The width and length of the microstrip line are denoted by *w*_1_ and *l*_1_, respectively. The width and length of the conversion structure are marked as *w*_2_ and *l*_2_, respectively. The width and length of HMSIW are marked as *w*_3_ and *l*_3_, respectively. Additionally, as shown in Fig. 1(a,b), the depths of the central grooves are the same, and the depths of the grooves on both sides decrease successively. The dimension of the central grooves is presented in Fig. 1(d), where the width, depth, and period of grooves are denoted by *a*, *h* and *p*_2_, respectively. Moreover, the diameter of the metallic via holes is *D* and the period length is *p*_1_, as shown in Fig. 1(c).

**Figure 2:**
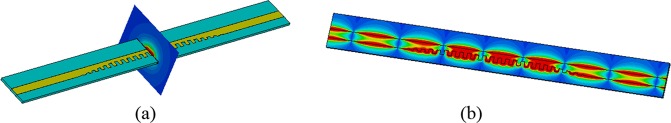
The simulated electric field distribution of the proposed band-pass filter on difference direction. (**a**) on the cross sections at 30 GHz; (**b**) on the x-y plane at 30 GHz.

In addition, the double layers SSPPs structure consists of two corrugated metal strips which etched on the upper and bottom metal of the substrate. In order to show the SSPPs propagation property of the structure, the simulated electric field distribution on the cross sections and *x-y* plane at 30 GHz are plotted in Fig. 2. It is seen that the EM fields are highly limited on the adjacent grooves and most of the EM energy is highly localized in the two-layer structure. Therefore, the structure can support the SSPPs mode propagation on its surface.

**Figure 3:**
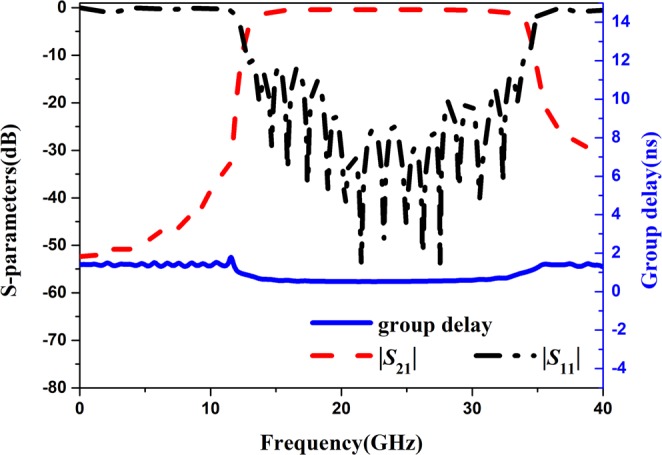
S-parameters and group delay of the proposed band-pass filter.

Figure 3 shows the S-parameters and group delay of the proposed band-pass filter. The group delay is an essential parameter for a wide band-pass filter and the value is the derivative of phase versus frequency. The physical meaning of group delay is to reflect the speed of the phase changing with frequency changing. The ideal state is that the group delay is a constant. It can be seen that the group delay of the proposed band-pass filter is approximately constant in the band-pass frequency range. It means that the phase of the transmitted signal does not produce distortion.

### Design consideration

#### Dispersion analysis of HMSIW, SSPPs, and the proposed band-pass filter

**Figure 4:**
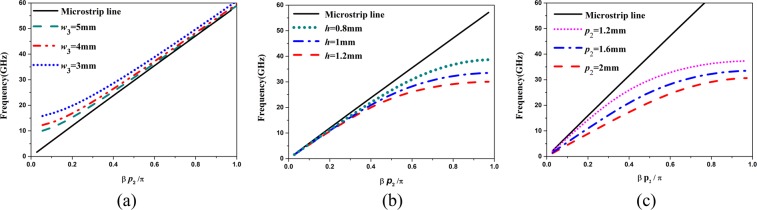
Simulated dispersion curves for (**a**) HMSIW with different width; (**b**) SSPPs with different groove depth; (**c**) SSPPs with different period.

**Figure 5:**
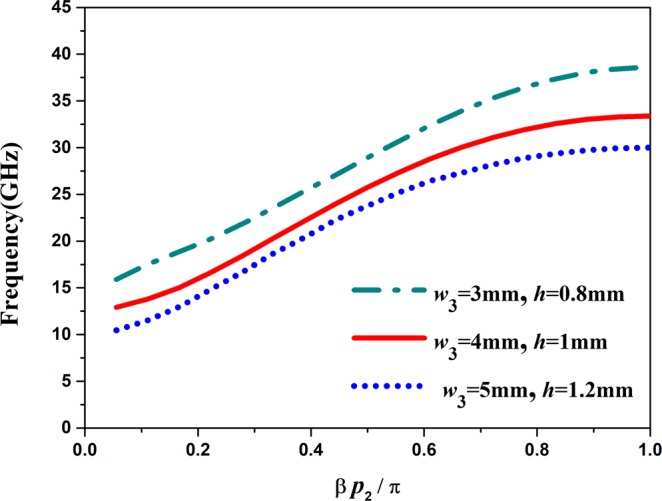
The dispersion curves of the proposed band-pass filter with different parameters.

In order to study the propagation characteristics of HMSIW, SSPPs, and the proposed band-pass filter, the dispersion characteristics are analyzed using the Eigen mode analysis of CST. The dispersion curves of HMSIW are portrayed in Fig. 4(a). It can be observed that HMSIW has a low cut-off frequency which increases with the decrease of width (*w*_3_) of HMSIW. Figure 4(b,c) show the dispersion curves of SSPPs. It can be seen that SSPPs have a high cut-off frequency which decreases as the depth (*h*) and period (*p*_2_) increases. The dispersion curves of the proposed band-pass filter with different parameters are shown in Fig. 5. The proposed band-pass filter, combining the propagation characteristics of HMSIW and SSPPs, has both low and high cut-off frequencies. In addition, the low and high cut-off frequencies can be independently controlled by changing corresponding parameters.

#### Parametric studies on the key parameter

**Figure 6:**
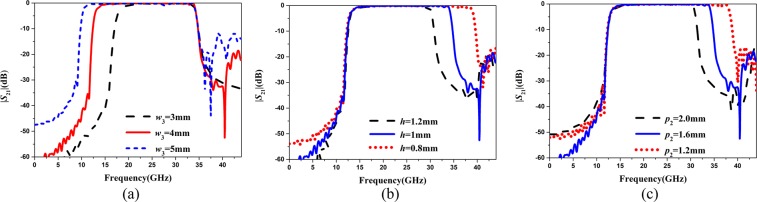
Simulated |*S*_21_| of the proposed band-pass filter with (**a**) difference *w*_3_; (**b**) difference *h*; (**c**) difference *p*_2_.

The bandwidth of the proposed band-pass filter is determined by the low and high cut-off frequencies, which can be controlled independently by tuning related parameters. In order to study the relationship between parameters and cut-off frequencies, the main parameters of the proposed band-pass filter are studied. Figure 6(a) shows the simulated transmission coefficients (|*S*_21_|)of the proposed band-pass filter with different *w*_3_. It turns out that the parameter *w*_3_ controls the low cut-off frequency, which decreases as the parameter raises. The simulated transmission coefficients (|*S*_21_|) of the proposed band-pass filter with difference *h* are shown in Fig. 6(b). It is seen that the high cut-off frequency can be controlled by the parameter *h*, which decreases as the *h* increases. As shown in Fig. 6(c), the *p*_2_ can also control the high cut-off frequency. The above analysis demonstrates that the proposed band-pass filter has a good controllability by tuning different *w*_3_, *h* and *p*_2_, respectively.

#### Equivalent circuit of the proposed band-pass filter

**Figure 7:**
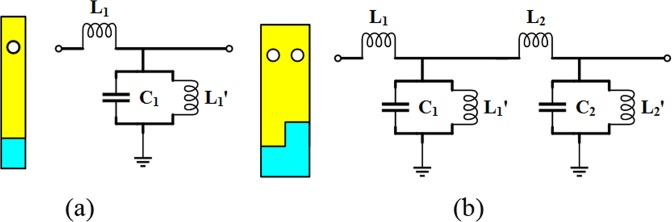
(**a**) The equivalent circuits of the HMSIW; (**b**) the equivalent circuits of unit cell of the proposed band-pass filter.

To further explain the structural behavior physically, we study the simplified LC equivalent circuit of HMSIW (ignoring the R value), as shown in Fig. 7(a), which is similar to a traditional rectangular waveguide^29–32^. The values of *L*_1_, *C*_1_ and *L*_1_’ can be expressed as:

1 $${L}_{1}=\frac{\pi t\mu \sqrt{1-(\lambda /2{w}_{3})}}{2{w}_{3}\sqrt{\mu /\varepsilon }},$$

2 $${C}_{1}=\frac{{{\varepsilon }}_{0}{{\varepsilon }}_{{r}}{{w}}_{3}{l}}{4{t}},$$

3 $${L^{\prime} }_{1}=\frac{1}{(2\pi {f}_{c}){C}_{1}}.$$

where *w*_3_, *t* are the width and thickness of the HMSIW, respectively. *f*_*c*_ is the cut-off frequency of the HMSIW and *l* is the length of the unit cell.

**Figure 8:**
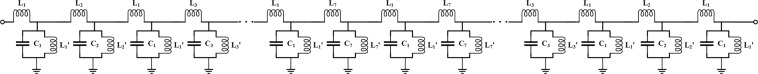
The equivalent circuits of the proposed band-pass filter.

**Figure 9:**
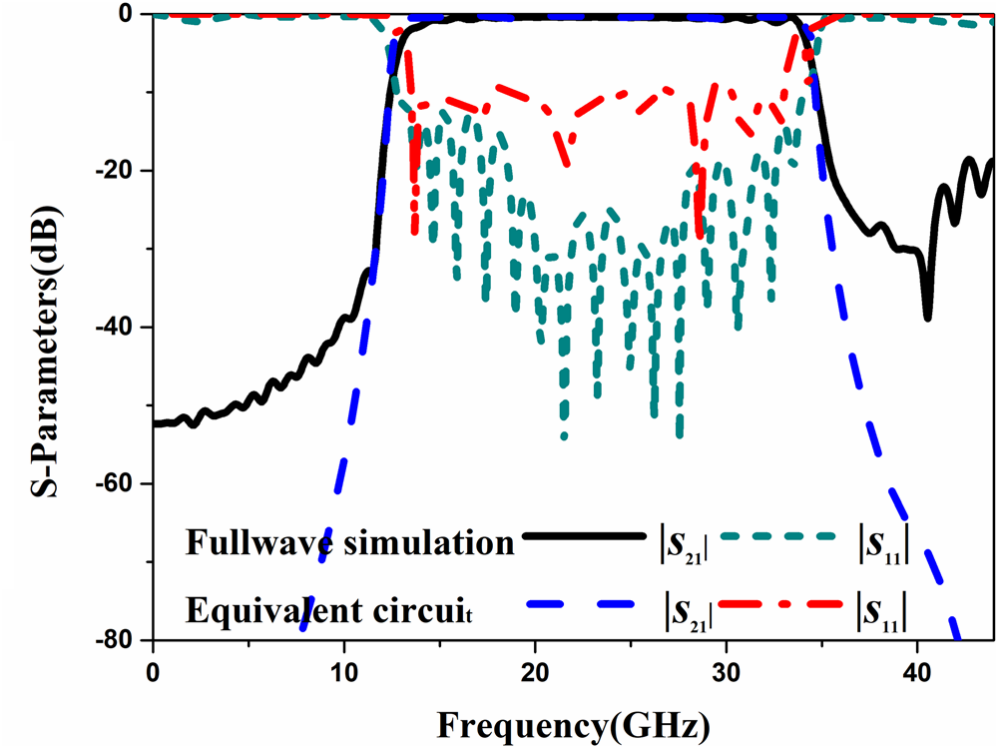
Simulated S-parameters of the proposed band-pass filter and equivalent circuit model.

Now, the equivalent circuit of the unit cell of the proposed band-pass filter is considered. This unit cell of the proposed band-pass filter can be viewed as HMSIW structures with different sizes in series, so the equivalent circuit model of unit cell is shown in Fig. 7(b). A simplified equivalent circuit model of the proposed band-pass filter is shown in Fig. 8. The LC values obtained from Eqs (1–3) are used as the initial values of optimization to get a good performance of the equivalent circuit. Since the coupling between the grooves is not considered in the equivalent process, a certain range of errors between the calculated value and the optimized value is present. The calculated values, optimized values and errors of the equivalent circuit are shown in Table 1. Figure 9 shows the simulation results of the proposed band-pass filter obtained by both full wave simulation and the extracted equivalent circuit model. It can be seen that the cut-off frequencies have a good agreement.

**Table 1:** The calculated, optimized values and errors of the equivalent circuit.

Symbol	Calculated (nH)	Optimized (nH)	Errors (%)	Symbol	Calculated (nH)	Optimized (nH)	Errors (%)		Calculated (pF)	Optimized (pF)	Errors (%)
L_1_	0.148	0.140	5.8	${{\rm{L}}^{\prime} }_{1}$	2.014	1.929	4.2	C_1_	0.0938	0.0817	12.9
L_2_	0.161	0.168	4.3	${{\rm{L}}^{\prime} }_{2}$	2.065	2.421	17.2	C_2_	0.0915	0.0825	9.8
L_3_	0.197	0.208	5.5	${{\rm{L}}^{\prime} }_{3}$	2.174	2.019	7.1	C_3_	0.0868	0.0993	14.4
L_4_	0.272	0.312	14.7	${{\rm{L}}^{\prime} }_{4}$	2.295	2.435	6.1	C_4_	0.0821	0.0754	8.1
L_5_	0.370	0.308	16.7	${{\rm{L}}^{\prime} }_{5}$	2.430	2.091	13.9	C_5_	0.0774	0.0746	3.6
L_6_	0.441	0.419	5.0	${{\rm{L}}^{\prime} }_{6}$	2.582	2.319	2.4	C_6_	0.0727	0.0631	13.2
L_7_	0.570	0.615	7.8	${{\rm{L}}^{\prime} }_{7}$	2.665	2.404	9.8	C_7_	0.0704	0.0733	4.1

### Experimental verification

**Figure 10:**
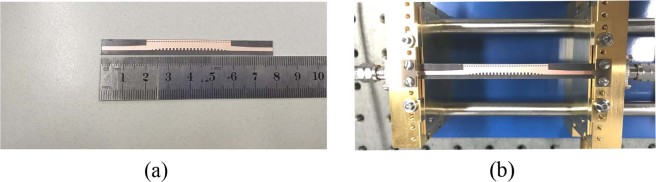
(**a**) Photograph of the fabricated band-pass filter; (**b**) measurement setup.

**Figure 11:**
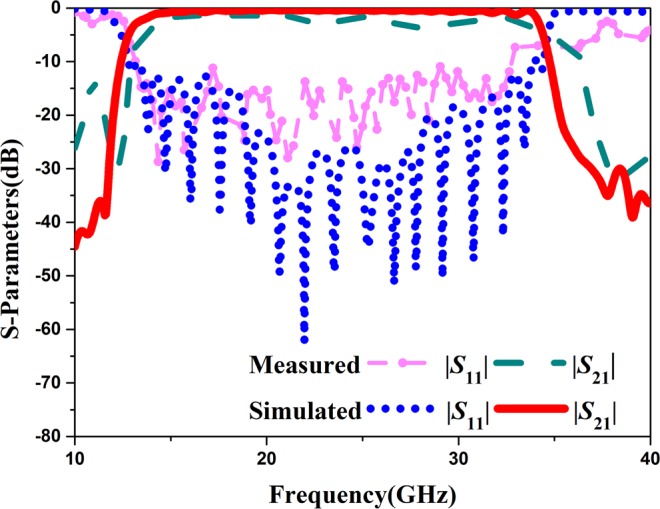
Measured and simulated S-parameters of the proposed band-pass filter.

We fabricate a prototype band-pass filter and measure its properties. The photograph of the fabricated band-pass filter is shown in Fig. 10(a). The structure is printed on the 0.5 mm thick F4B substrate with relative permittivity *ε*_*r*_ = 2.65, loss tangent tan *δ* = 0.0015, and the copper layer has a thickness of 0.018 mm. The physical dimensions of the fabricated band-pass filter are as follows: *l*_1_ = *l*_2_ = 10 mm, *l*_3_ = 39.2 mm, *a* = 0.8 mm, *h* = 1 mm, *w*_1_ = 1.8 mm, *w*_2_ = 2.25 mm, *w*_3_ = 4 mm, *p*_1_ = 0.8 mm and *p*_2 =_1.6 mm. The proposed band-pass filter is measured with the setup shown in Fig. 10(b). Figure 11 presents the simulated and measured results of the proposed band-pass filter. Simulated results show that the proposed band-pass filter achieves a bandwidth (for |*S*_11_| < −10dB and |*S*_21_| > −0.8 dB) from 15.6 to 32.1 GHz. It is seen that the measured results are in good agreements with the simulated ones at the band-pass frequencies. But the measured return loss and insertion loss are a little greater than the simulated results at high frequency that maybe because of the errors of fabricated process and measured tools. Table 2 compares the proposed band-pass filter with some previous hybrid SIW and SSPPs filters in terms of simulated performances, where λ is the wavelength at the lowest frequency. It is seen that the band-pass filters in^26^ and^28^ have a smaller thickness, but their transmission efficiency needs to be improved. The filters in^7^ and^27^ have a high efficiency, but these have wide width or thick substrate. Therefore, compared to other band-pass filters, the proposed band-pass filter has smaller size, wider bandwidth, and lower loss.

**Table 2:** Simulated performance comparison of existing band-pass filter.

Work	Dimension (λ) Width × Thickness	Bandwidth (GHz)	Return Loss (dB)	Insertion Loss (dB)	Type
Ref.^7^	0.79 × 0.021	11.92–21.54 (57.5%)	10	1	hybrid SIW and SSPPs
Ref.^26^	0.49 × 0.012	7.3–11.2 (44%)	12	2	hybrid SIW and SSPPs
Ref.^27^	(not given) × 0.11	16.1–25.4 (43.8%)	10	1.02	hybrid SIW and SSPPs
Ref.^28^	0.58 × 0.016	9.74–16.7 (52.65%)	10	1.5	hybrid HMSIW and SSPPs
This work	0.36 × 0.025	15.6–32.1 (69.77%)	10	0.8	hybrid HMSIW and SSPPs

## Discussion

In this paper, we proposed a controllable band-pass filter based on HMSIW and double-layer SSPPs, which can realize band-pass transmission by etching periodic grooves at the top and bottom metal layers of HMSIW. The dispersion characteristics and parametric studies of the proposed band-pass filter have been analyzed. The simulated results validated that the proposed band-pass filter had a wider bandwidth and higher transmission efficiency. Moreover, by changing the width of the HMSIW, the depth and period of the periodic grooves, the cut-off frequencies of the proposed band-pass filter could be controlled independently. Finally, a sample of the proposed band-pass filter was fabricated, and the measured results were in good agreement with the simulated ones, verifying that the proposed band-pass filter has a good performance and may be used in microwave frequencies for higher integrations.

## Methods

By using the commercial software, CST Microwave Studio, we studied the dispersion relations of SSPPs, HMSIW, S-parameters and surface fields of the filter. As shown in Fig. 10(a), a prototype band-pass filter is fabricated using 0.5 mm thick F4B substrate with relative permittivity *ε*_*r*_ = 2.65, and loss tangent tan *δ* = 0.0015. The copper layer has a thickness of 0.018 mm. The Vector Network Analyzer was used to measure the S-parameters of the fabricated samples.

## References

[1] Deslandes D, Wu K (2001). Integrated Microstrip and Rectangular Waveguide in Planar Form. IEEE Microw. Wireless Compon. Lett..

[2] Bahar AAM (2019). Real Time Microwave Biochemical Sensor Based on Circular SIW Approach for Aqueous Dielectric Detection. Sci. Rep..

[3] Xu F, Wu K (2005). Guided-Wave and Leakage Characteristics of Substrate Integrated Waveguide. IEEE Trans. Microwave Theory Tech..

[4] Hong, W., Liu, B., Wang, Y.Q., Lai, Q. H. & Wu, K. Half mode substrate integrated waveguide: A new guided wave structure for microwave and millimeter wave application, In Proc. Joint 31st Int. Infrared Millimeter Wave Conf. 14th Int. *Terahertz Electron. Conf*. Shanghai, China. 18–22 (2006).

[5] Lai Q, Fumeaux C, Hong W, Vahldieck R (2009). Characterization of the propagation properties of the half-mode substrate integrated waveguide. IEEE Trans. Microwave Theory Tech..

[6] Song KJ, Zhu Y, Zhang F (2017). Single- and dual-band filtering-response power dividers embedded SIW filter with improved output isolation. Sci. Rep..

[7] Zhang Q, Zhang HC, Wu H, Cui TJ (2015). A Hybrid Circuit for Spoof Surface Plasmons and Spatial Waveguide Modes to Reach Controllable Band-Pass Filters. Sci. Rep..

[8] Fan C, Choi WW, Yang WC, Che WQ, Tam KW (2016). A Low Cross-Polarization Reflectarray Antenna Based on SIW Slot Antenna. IEEE Antennas Wireless Propag. Lett..

[9] Li XP, Xiao J, Qi ZH, Zhu H (2018). Broadband and High-Gain SIW-Fed Antenna Array for 5G Applications. IEEE Access..

[10] Pendry JB, Martin-Moreno L, Garcia-Vidal FJ (2004). Mimicking Surface Plasmons with Structured Suifaces. Science.

[11] Garcia-Vidal FJ, Martin-Moreno L, Pendry JB (2005). Surfaces with holes in them: New plasmonic metamaterials. J. Opt. A: Pure Appl. Opt..

[12] Hibbins AP, Evans BR, Sambles JR (2005). Experimental Verification of Design Surface Plasmons. Science.

[13] Wang J, Zhao L, Hao ZC (2019). A Band-Pass Filter Based on the Spoof Surface Plasmon Polaritons and CPW-Based Coupling Structure. IEEE Access..

[14] Gao X, Che W, Feng W (2018). Novel non-periodic spoof surface plasmon polaritons with H-shaped cells and its application to high selectivity wideband bandpass filter. Sci. Rep..

[15] Hao ZC, Zhang JX, Zhao L (2019). A compact leaky-wave antenna using a planar spoof surface plasmon polariton structure. International Journal of RF and Microwave Computer-Aided Engineering..

[16] Liu JS, Ding L, Wang KJ, Yao JQ (2009). A method to design transmission resonances through subwavelength apertures based on designed surface plasmons. Opt. Express..

[17] Jiang T, Shen L, Zhang X, Ran L (2009). High-order modes of spoof surface plasmon polaritons on periodically corrugated metal surfaces. Prog. Electromagn. Res..

[18] Wang J, Zhao L, Hao ZC, Cui TJ (2018). An ultra-thin coplanar waveguide filter based on the spoof surface plasmon polaritons. Appl. Phys. Lett..

[19] Tian L, Liu J, Zhou K, Gao Y, Liu S (2016). Investigation of mechanism: spoof SPPs on periodically textured metal surface with pyramidal grooves. Sci. Rep..

[20] Schnitzer O (2017). Spoof surface plasmons guided by narrow grooves. Phys. Rev. B..

[21] Shen LF, Chen XD, Zhang XF, Agarwal K (2011). Guiding Terahertz Waves by a Single Row of Periodic Holes on a Planar Metal Surface. Plasmonics..

[22] Zhao L, Liu S, Wang J, Shen XP, Cui TJ (2019). A Band-stop filter based on spoof surface plasmon polartion. Electronics Letters..

[23] Xu Z, Li S, Yin X, Zhao H, Liu L (2017). Radiation loss of planar surface plasmon polaritons transmission lines at microwave frequencies. Sci. Rep..

[24] Zhao L (2016). A Novel Broadband Band-pass Filter Based on Spoof Surface Plasmon Polartons. Sci. Rep..

[25] Zhang HC, Cui TJ, Zhang Q, Fan YF, Fu XJ (2015). Breaking the Challenge of Signal Integrity Using Time-Doman Spoof Surface Plasmon Polaritons. Acs Photonics..

[26] Chen P, Li LP, Yang K, Chen Q (2018). Hybrid Spoof Surface Plasmon Polariton andSubstrate Integrated Waveguide Broadband Bandpass Filter With Wide Out-of-Band Rejection. IEEE Microw. Wireless Compon. Lett..

[27] Guan DF, You P, Zhang QF, Xiao K, Yong SW (2017). Hybrid Spoof Surface Plasmon Polariton and Substrate Integrated Waveguide Transmission Line and Its Application in Filter. IEEE Trans. Microwave Theory Tech..

[28] Guan DF (2018). Slow-Wave Half-Mode Substrate Integrated Waveguide Using Spoof SurfacePlasmon Polariton Structure. IEEE Trans. Microwave Theory Tech..

[29] Hrabar S, Bartolic J, Sipus Z (2005). Waveguide miniaturization using uniaxial negative permeability metamaterial. IEEE Trans. Antennas Propag..

[30] Dhwaj K, Kovitz JM, Tian HZ, Jiang LJ, Itoh T (2018). Half-Mode Cavity-Based Planar Filtering Antenna with Controllable Transmission Zeroes. IEEE Antennas Wireless Propag. Lett..

[31] Dong YD, Tatsuo I (2010). Miniaturized Substrate Integrated Waveguide Slot Antennas Based on Negative Order Resonance. IEEE Trans. Antennas Propag..

[32] Dong YD, Tatsuo I (2010). Composite Right/Left-Handed Substrate Integrated Waveguide and Half Mode Substrate Integrated Waveguide Leaky-Wave Structures. IEEE Trans. Antennas Propag..

